# ROS‐Mediated PARP Activity Undermines Mitochondrial Function After Permeability Transition Pore Opening During Myocardial Ischemia–Reperfusion

**DOI:** 10.1161/JAHA.113.000159

**Published:** 2013-04-24

**Authors:** Jacqueline M. Schriewer, Clara Bien Peek, Joseph Bass, Paul T. Schumacker

**Affiliations:** 1 Division of Neonatology, Department of Pediatrics, Northwestern University Feinberg School of Medicine, Chicago, IL (J.M.S., P.T.S.); 2 Division of Endocrinology, Metabolism, and Molecular Medicine, Department of Medicine, Northwestern University Feinberg School of Medicine, Chicago, IL (C.B.P., J.B.)

**Keywords:** cell death, heart, mitochondrial potential, myocardial infarction, oxidant stress

## Abstract

**Background:**

Ischemia–reperfusion (I/R) studies have implicated oxidant stress, the mitochondrial permeability transition pore (mPTP), and poly(ADP‐ribose) polymerase (PARP) as contributing factors in myocardial cell death. However, the interdependence of these factors in the intact, blood‐perfused heart is not known. We therefore wanted to determine whether oxidant stress, mPTP opening, and PARP activity contribute to the same death pathway after myocardial I/R.

**Methods and Results:**

A murine left anterior descending coronary artery (LAD) occlusion (30 minutes) and release (1 to 4 hours) model was employed. Experimental groups included controls and antioxidant‐treated, mPTP‐inhibited, or PARP‐inhibited hearts. Antioxidant treatment prevented oxidative damage, mPTP opening, ATP depletion, and PARP activity, placing oxidant stress as the proximal death trigger. Genetic deletion of cyclophilin D (CypD^−/−^) prevented loss of total NAD^+^ and PARP activity, and mPTP‐mediated loss of mitochondrial function. Control hearts showed progressive mitochondrial depolarization and loss of ATP from 1.5 to 4 hours of reperfusion, but not outer mitochondrial membrane rupture. Neither genetic deletion of PARP‐1 nor its pharmacological inhibition prevented the initial mPTP‐mediated depolarization or loss of ATP, but PARP ablation did allow mitochondrial recovery by 4 hours of reperfusion.

**Conclusions:**

These results indicate that oxidant stress, the mPTP, and PARP activity contribute to a single death pathway after I/R in the heart. PARP activation undermines cell survival by preventing mitochondrial recovery after mPTP opening early in reperfusion. This suggests that PARP‐mediated prolongation of mitochondrial depolarization contributes significantly to cell death via an energetic crisis rather than by mitochondrial outer membrane rupture.

## Introduction

Myocardial ischemia is a leading cause of death in the Western world.^1^ Despite intensive study, the molecular mechanisms contributing to cell death in ischemia–reperfusion (I/R) injury are not fully understood.^2–3^ Cell death after I/R is characterized by loss of mitochondrial function, bioenergetic failure, and eventual loss of plasma membrane integrity. Studies have identified oxidant stress,^4–5^ opening of the mitochondrial permeability transition pore (mPTP),^6^ and excessive activation of the DNA repair enzyme poly(ADP‐ribose) polymerase (PARP)^7^ as contributing factors in I/R injury. Although each of these elements has been linked to loss of mitochondrial function and eventual cell death, no single study has determined the relationships and interdependence among these factors during myocardial I/R.

Previous work has revealed that mitochondrial oxidant stress is an important early mediator of I/R injury.^2,8^ Reactive oxygen species (ROS) are generated during ischemia and increase significantly within minutes after reperfusion, contributing to membrane lipid peroxidation, oxidative changes in protein structure/function, and oxidative damage to DNA.^9–12^ Accordingly, significant protection from I/R injury is conferred in numerous models by overexpression of antioxidant enzymes targeted to the mitochondria or by broad‐spectrum antioxidants administered at or before reperfusion.^5,12–13^ Although exogenous administration of oxidants can certainly trigger cell death, it is not known whether I/R‐induced oxidants trigger lethal nonspecific membrane rupture or whether they act by initiating downstream pathways that lead to cell death.

In addition to creating oxidant stress, reperfusion induces opening of the mitochondrial permeability transition pore (mPTP), a small yet undefined channel within the inner mitochondrial membrane.^14^ Opening of the mPTP depolarizes the mitochondria and allows the redistribution of solutes smaller than 1.5 kDa, such as NAD(H) and calcium, between the matrix and the cytosol.^15–16^ Although transient opening of the mPTP may occur in normal cells, sustained opening of the mPTP appears to induce cell death by increasing oxidant stress, causing ATP depletion, and/or by triggering matrix swelling and subsequent rupture of the outer mitochondrial membrane.^17^ However, the precise mechanism and timing of cell death induced by mPTP opening in the intact, blood‐perfused heart has not been established.

Strong evidence indicates that mPTP opening is enhanced by oxidative stress,^18–21^ although other findings suggest that mPTP opening can itself induce oxidant generation.^22^ Mice carrying homozygous deletion of the gene encoding cyclophilin D, a regulator of the mPTP, are protected from I/R injury and are more resistant to exogenous oxidant stress, suggesting that the mPTP can mediate cell death caused by an oxidant challenge.^6,14,23–24^ However, other studies suggest that mPTP opening may enhance oxidant stress by increasing superoxide generation from futile cycling of the mitochondrial electron transport system.^22,25^ Hence, the interdependence between mPTP opening and oxidative stress in the reperfused myocardium is not fully understood.

A third mediator of cell death after I/R injury is poly(ADP‐ribose) polymerase‐1 (PARP).^26^ Under conditions of increased DNA damage, activated PARP consumes NAD^+^ as it facilitates nuclear DNA repair,^27–28^ but its hyperactivation has been linked to mitochondria‐mediated necrosis.^29^ As with the mPTP, the proposed mechanisms of cell death mediated by excessive PARP activity include the enhancement of oxidant generation, bioenergetic crisis arising from the depletion of NAD^+^ and ATP, and disruption of the outer mitochondrial membrane allowing release of prodeath proteins such as apoptosis‐inducing factor (AIF).^30–33^ Chemical inhibitors of PARP, or the genetic deletion of PARP‐1, confer protection against I/R injury in the myocardium as well as against exogenous oxidant challenge.^7,34^ Although PARP activation can be caused by oxidative stress,^34^ its activation may also trigger the generation of oxidants.^30,35^ Increases in oxidant stress have been linked to poly(ADP‐ribosylation) of mitochondrial proteins after I/R,^36^ which could contribute to mitochondrial ROS generation. This suggests that PARP activity could mediate cell death caused by oxidative damage after I/R, and it could also promote cell death by amplifying oxidant stress. As with mPTP studies, it is unclear whether significant oxidative damage is required for, or the product of, PARP activity in the reperfused myocardium.

The observation that inhibition of either PARP or the mPTP is protective in I/R injury suggests that they may be connected mechanistically. For example, PARP could trigger mPTP activation by poly(ADP‐ribosylation) of a mitochondrial target or by enhancing oxidant generation. Conversely, mPTP opening could activate PARP by increasing oxidant stress, or it could facilitate PARP activity by releasing NAD^+^ from the mitochondria to the cytosol. Importantly, it is not known whether opening of the mPTP is required for PARP activity, whether PARP activity opens the mPTP, or whether opening of the mPTP is a terminal event in a separate pathway from PARP‐induced myocardial cell death.

Previous studies have partially examined the relationships among oxidative stress, mPTP function, and PARP activity using simulated stress conditions, buffer‐perfused models, or cell‐based models of simulated I/R.^19,25,30,37–38^ However, no study has examined the interdependence of these factors using an in vivo model of left anterior descending coronary artery (LAD) occlusion and reperfusion coupled with a biochemical analysis. The present study therefore sought to dissect interactions among oxidative stress, the mPTP, and PARP activity in a single in vivo model of myocardial I/R in the same genetic background.

## Methods

### Animals

All aspects of animal care and experimentation were performed in accordance with the Institutional Animal Care and Use Committee. C57BL/6N mice were purchased from Charles River (Wilmington, MA). Mice lacking the cyclophilin D gene (Ppif^−/−^)^24^ were back‐bred by 9 generations into a C57BL/6 background. Mice lacking the gene *PARP‐1* were purchased from Jackson Labs (Bar Harbor, ME) and were back‐bred into the C57BL/6 lineage by at least 4 generations. Heterozygous breedings yielded PARP‐1‐null animals and wild‐type littermate controls. Mice were fed rodent chow ad libitum and housed in a facility with a 12‐hour light/dark cycle.

### LAD Occlusion

LAD occlusion experiments were carried out on adult (8 to 11 weeks) male mice. Anesthesia was induced by an intraperitoneal injection of tri‐bromoethanol (Avertin, 200 mg/kg) and maintained with supplementary doses (25 mg/kg) as needed to prevent the hind limb withdrawal reflex. Rectal temperature was maintained at 37.2±0.5°C. Mice were intubated with a 20‐gauge×1 inch cannula (BD) by direct visualization and ventilated with 100% O_2_ using a pressure‐controlled rodent ventilator (Kent Scientific) at 140 breaths per minute with a maximal inspiration pressure of 16 cm H_2_O and a positive‐end expiratory pressure of 4 cm H_2_O. Mice were taped to a heated surgical table in the supine position. Hair was removed from the left thorax with depilatory cream. The chest was then opened using an intercostal incision at the third intercostal space, and blood vessels were cauterized to minimize blood loss. The pericardium was bluntly dissected away and draped over the left lung. The LAD was visualized as a bright red vessel coursing along the left ventricular epicardium, stemming from the aortic root just under the left atria. A 6‐0 silk suture was passed under the LAD ≈1 to 2 mm from the tip of the left atrium. Tension was gently applied to the suture to occlude the LAD, and the occlusion was maintained with a small bulldog clamp. Ischemia was maintained for 30 minutes, and reperfusion was achieved by removal of the bulldog clamp and confirmed by visual inspection. A 22‐gauge×1 inch cannula was threaded through the skin and fourth intercostal space and was placed between the heart and left lung. The thorax was then closed with 4‐0 silk suture; the skin was securely closed with a running stitch. Trapped air was released by putting gentle pressure on the rib cage; a negative pleural pressure was restored by applying gentle suction through the 22‐gauge cannula. Mice were disconnected from the ventilator, extubated, and allowed to recover under mild anesthesia while rectal temperature was maintained at 37°C. Mice were then placed in a cage with an ambient temperature of 32°C. Reperfusion was allowed to progress for 4 or 16 hours before histological analysis. In some studies mice were maintained on mechanical ventilation during reperfusion for 15‐minute, 1‐hour, or 1.5‐hour periods, at which times the hearts were harvested for biochemical analysis.

### Injectable Drugs

Drugs were dissolved in sterile saline. EUK134 (EUK, 10 mg/kg; Cayman Chemicals), a SODII and catalase mimetic, was injected retro‐orbitally during anesthesia prior to the start of surgery. The PARP inhibitor 3‐aminobenzamide (3AB, 20 mg/kg; Sigma‐Aldrich) was injected retro‐orbitally prior to the start of surgery and supplemented (10 mg/kg IP) 1 hour after reperfusion.

### Infarct Analysis

At the end of the designated reperfusion period, the LAD was reoccluded, and 0.5% MTT dye (Sigma‐Aldrich) was injected into the abdominal aorta, retroperfusing nonischemic tissue and staining it dark purple. Hearts were then excised and cut into 1‐mm sections using a heart matrix (Zivic Instruments) and placed into 1% TTC (Sigma‐Aldrich) until the sections stained brick red (≈10 minutes). Sections were then fixed overnight in 3.7% formaldehyde. Both sides of each slice were then photographed, the right ventricle was trimmed away, and the slices were weighed. The area of each region of interest was determined by planimetry using ImageJ software (NIH). For each slice, the areas of interest were measured on each side and averaged. The volume of affected tissue was then calculated by multiplying the fractional area of interest by the weight of the slice. These volumes were then summed across slices to yield total volumes of affected tissue. The area at risk (AAR) was defined as the volume of tissue that stained red or white relative to the entire volume of the LV and septum. The degree of necrosis was defined as the volume of tissue stained white divided by the volume of the area at risk as previously described.^39^

### Mitochondrial Polarization Analysis

Tetramethylrodamine ethyl ester (TMRE) was injected retro‐orbitally (4 mg/kg) 15 minutes before analysis. Following reperfusion, the LAD was reoccluded, and Hoechst 33342 dye was retroperfused from the aorta into the nonischemic tissue. Hearts were then excised, cut into 1‐mm sections, and immediately imaged on a dissecting microscope with fluorescence visualization capability. For calcein control studies, Hoechst was perfused, and hearts were excised, sliced, and placed into calcein‐AM (20 μmol/L) for 15 minutes prior to imaging. Fluorescence images were captured with a Nikon AZ‐100 using the 451‐nm (Hoescht), 512‐nm (Calcein), and 570‐nm (TMRE) emission filters. Average fluorescence of the AAR relative to the LV was analyzed using Metamorph software (Invitrogen) by tracing regions of interest in the Hoechst channel and measuring the average fluorescence of those regions in the TMRE or calcein channel. Fluorescence ratios were compared to a sham control.

### Subcellular Fractionation

Subcellular fractionation of the area at risk was performed using a Mitochondria Isolation Kit for Tissue (Thermo Scientific). The AAR was labeled by reoccluding the LAD and infusing 300 μL of 0.5% MTT dye into the heart through a left ventricular puncture. The AAR, as defined by tissue that did not stain purple, was then excised and placed into ice‐cold PBS. Tissue was minced in PBS containing 1 mg/mL trypsin and allowed to incubate on ice for 5 minutes. Heart pieces were pelleted, and trypsin solution was replaced with 800 μL of Buffer A containing 4 mg/mL fatty acid–free BSA, and protease inhibitors. Heart pieces were homogenized with a 2‐mL Dounce homogenizer for 10 strokes with pestle A and 15 strokes with pestle B. Homogenates were then added to 800 μL of Buffer C with protease inhibitors. Nuclei and small amounts of unhomogenized tissue were pelleted at 700*g* for 10 minutes. Supernatants were placed into a new tube, and cytosolic and mitochondrial fractions were separated by differential centrifugation at 6000*g* for 15 minutes. Cytosolic fractions were used for Western analysis. Mitochondrial pellets were immediately deproteinated in 0.5 mol/L perchloric acid.

### Adenine Nucleotide Determination

Quantification of NAD^+^ and ATP levels from flash‐frozen tissue and mitochondrial fractions was performed on neutralized 0.5 mol/L perchloric acid extracts. Total adenine nucleotide concentration was measured in tissue that had been freeze‐clamped in vivo with liquid nitrogen–cooled tongs, perchloric acid precipitated, and compared with total protein. After removing precipitated proteins by centrifugation, supernatants were neutralized with potassium hydroxide (3 mol/L), and NAD^+^ was measured using a cycling reaction as previously described.^40^ NAD^+^ concentrations were determined by comparison to a standard curve and normalized to protein concentration as determined by a BCA Protein Kit (Pierce Scientific). ATP, ADP, and AMP levels were assessed by high‐performance liquid chromatography with Waters 515 pumps and a 2487 detector with a Supelco LC‐18‐T column (15×4.6 cm). ATP, ADP, and AMP eluted as sharp peaks and were quantified by comparison with a standard curve and normalized to the protein content of extracted tissue.

### Western Blot

Proteins were denatured in SDS loading buffer, boiled, subjected to SDS‐PAGE, and Western‐blotted. Proteins were visualized by enhanced chemiluminescence according to the manufacturer's instructions (Amersham Biosciences).

### Mitochondrial and Nuclear DNA Damage

Mitochondrial or nuclear DNA damage was measured as described previously.^41–42^ Genomic DNA was prepared from the AAR of reperfused tissue using Qiagen Genomic Tip and Buffer Kits. Long PCR products from the mitochondrial DNA (10 kb) and nuclear DNA (6.5 kb) were produced from equal quantities of genomic sample and stopped in their linear phase of amplification. A short fragment of mitochondrial DNA (117 bp) was used as a control for mitochondria copy number. PCR products were quantified using PicoGreen and visualized by ethidium bromide staining on an agarose gel.

### Total Protein Oxidation

Protein carbonylation was determined using a OxyBlot Protein Oxidation Detection Kit according to the manufacturer's instructions (Millipore). Briefly, 10 μg of total protein carbonyls was derivitized with dinitrophenylhydrazine (DNP) or control solution for 10 minutes. Reactions were stopped with neutralization solution, and proteins were dot‐blotted onto polyvinylidene fluoride membranes. Equal loading was confirmed by Ponceau stain. Proteins were then blotted with an anti‐DNP antibody and quantified by densitometry.

### Data Analysis

Data are expressed as means±standard errors of the mean. One‐way analysis of variance (ANOVA) was performed using GraphPad InStat software on data sets that passed normality tests followed by Student–Newman–Keuls post hoc tests to evaluate significant differences between groups. For analysis of data with multiple interventions, 2‐way ANOVA was performed using GraphPad Prism software to analyze potential interactions among groups, followed by Student–Newman–Keuls post hoc tests to evaluate significant differences. Additional analysis of data sets that failed normality testing was carried out using the Kruskal–Wallis test followed by the Dunn post hoc test to evaluate significance.

## Results

### Oxidant Stress, mPTP, and PARP All Contribute to Cell Death in Ischemia–Reperfusion Injury

**Figure 1.**
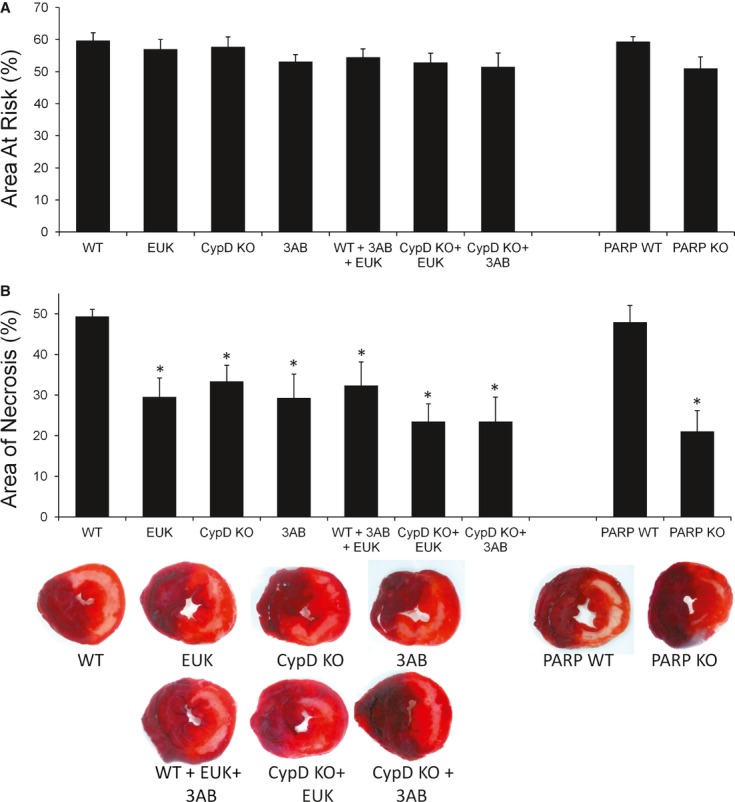
Identification of the area at risk (AAR) and the area of necrosis after I/R. A, AAR in wild‐type (WT) and experimental groups subjected to ischemia (30 minutes) followed by 4 hours of reperfusion: WT, n=8; EUK, n=6; CypD, n=6; 3AB, n=6; WT/3AB/EUK, n=6; CypD/3AB, n=7; CypD/EUK, n=8; PARP WT, n=6; PARP KO, n=6. No significant differences were detected. B, Area of necrosis in WT and experimental groups after ischemia (30 minutes) followed by 4 hours reperfusion. Representative slices from each group showing the LV not at risk (purple), the AAR (red+white), and the area of necrosis (white). *P<0.05 compared with WT. Values are means±SEMs. I/R indicates ischemia–reperfusion; EUK, EUK134, SODII, and catalase mimetic; CypD, cyclophilin D; 3AB, 3‐aminobenzamide; PARP, poly(ADP‐ribose) polymerase; KO, knockout; LV, left ventricle.

Although previous studies have examined the contributions of oxidant stress, mPTP opening, and PARP to I/R injury, no single study has compared their relative contributions to cell death in an in vivo model of myocardial ischemia–reperfusion (I/R) injury.^4,6–7^ We therefore tested whether single interventions with an antioxidant (EUK), genetic knockout of the mPTP modulator cyclophilin D (CypD KO), PARP inhibition with an inhibitor (3‐aminobenzamide [3AB]), or genetic knockout of PARP‐1 (PARP‐1 KO) conferred protection against I/R injury and whether double interventions with 3AB and EUK in CypD‐KO or WT hearts provided additive protection. An LAD occlusion model was used to induce ischemia for 30 minutes followed by 4 hours of reperfusion. Analysis confirmed that the area at risk (AAR) in the left ventricle did not differ among groups (Figure 1A). Animals treated with the antioxidant EUK or the PARP inhibitor 3AB exhibited significantly less necrotic staining compared with WT hearts. Similarly, mice with genetic deletion of CypD or PARP‐1 were also protected compared with isogenic WT control hearts (Figure 1B). Furthermore, WT or CypD‐KO mice treated with 3AB and/or EUK prior to I/R did not exhibit significant additive protection against I/R injury (Figure 1B). These results confirmed that ROS, opening of the mPTP, and PARP activity each contribute significantly to cell death in a single model of in vivo I/R. The lack of statistical interaction, either additive or negative, among groups with multiple interventions suggests that they may all function in a single pathway.

### Antioxidants Inhibit I/R‐Induced DNA Damage, But CypD KO, PARP‐1 KO, or 3AB Does Not

**Figure 2.**
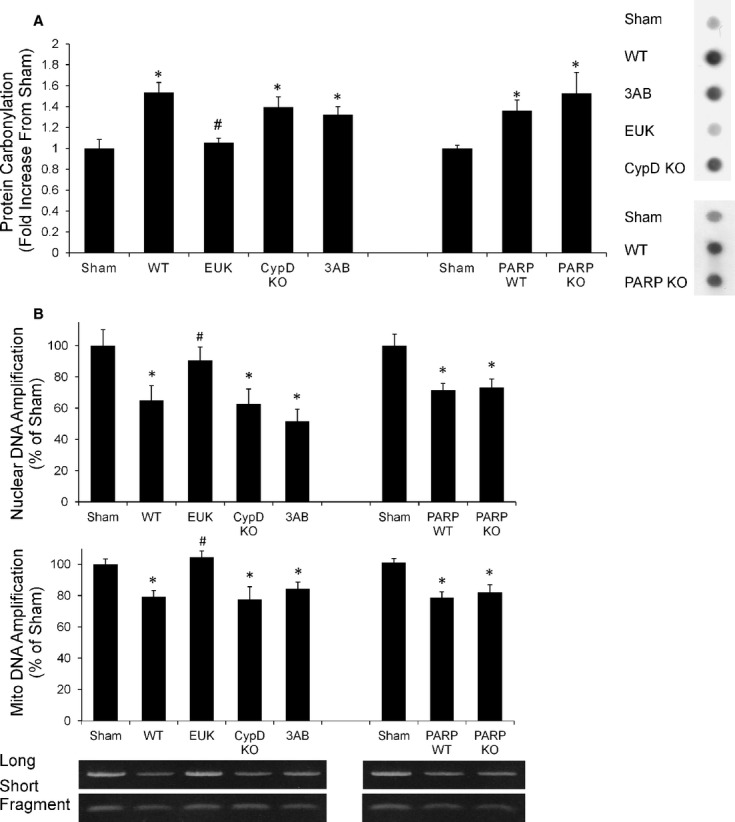
Protein oxidation and DNA damage after I/R. A, Analysis of dot blots of total cell protein from the AAR after derivatization of protein carbonyls. Representative dots from a panel blot are shown: sham, n=9; WT, n=8; EUK, n=7; CypD, n=9; 3AB, n=7; PARP WT, n=7; PARP KO, n=5. B, Nuclear and mitochondrial DNA amplification in sham, WT, and experimental groups after ischemia (30 minutes) followed by reperfusion for 90 minutes: sham, n=8; WT, n=6; EUK, n=5; CypD, n=5; 3AB, n=5; PARP WT, n=7; PARP KO, n=7. Values expressed relative to sham. Representative ethidium bromide–stained gels of the long and short mitochondrial products are shown. *P<0.05 compared with sham, # different compared with WT. Values are means±SEMs. I/R indicates ischemia–reperfusion; AAR, area at risk; WT, wild type; EUK, EUK134, SODII, and catalase mimetic; CypD, cyclophilin D; 3AB, 3‐aminobenzamide; PARP, poly(ADP‐ribose) polymerase; KO, knockout.

Oxidative stress has been shown to be both a cause and a consequence of mPTP opening and PARP activation.^19,22,30,34^ To test whether mPTP opening or PARP activity is required for significant oxidative damage after I/R, we assessed total protein oxidation and DNA damage as markers of oxidative stress in the nucleus and mitochondria.^43^ Total cellular protein was harvested from the AAR of hearts after 60 minutes of reperfusion and subjected to dot blots for total protein carbonylation. Protein oxidation was evident in WT, CypD‐KO, 3AB‐treated, and PARP‐1 KO samples, but not in EUK‐treated samples (Figure 2A). In addition, total cellular DNA was harvested 90 minutes into reperfusion from hearts treated with EUK or 3AB or lacking CypD or PARP‐1. Mitochondrial and nuclear DNA damage was analyzed by quantitative PCR. The presence of DNA damage led to a significant decrease in the PCR product of a long DNA segment compared with a short DNA segment, as previously shown.^42^ DNA from hearts harvested 90 minutes into reperfusion demonstrated less relative PCR amplification compared with sham samples for both nuclear and mitochondrial DNA amplification reactions, indicating the presence of DNA damage in the mitochondria and the nucleus. EUK treatment significantly attenuated this injury, confirming that this damage is the result of oxidant stress. DNA from hearts lacking CypD or PARP‐1 or inhibited with 3AB showed decreased amplification, indicating damage (Figure 2B). Taken together these results demonstrate that significant ROS‐mediated damage is evident even in the absence of mPTP opening and PARP activity after I/R.

### Oxidant Stress, mPTP Opening, and PARP Activity All Contribute to Protein Poly(ADP‐Ribosylation) in I/R

**Figure 3.**
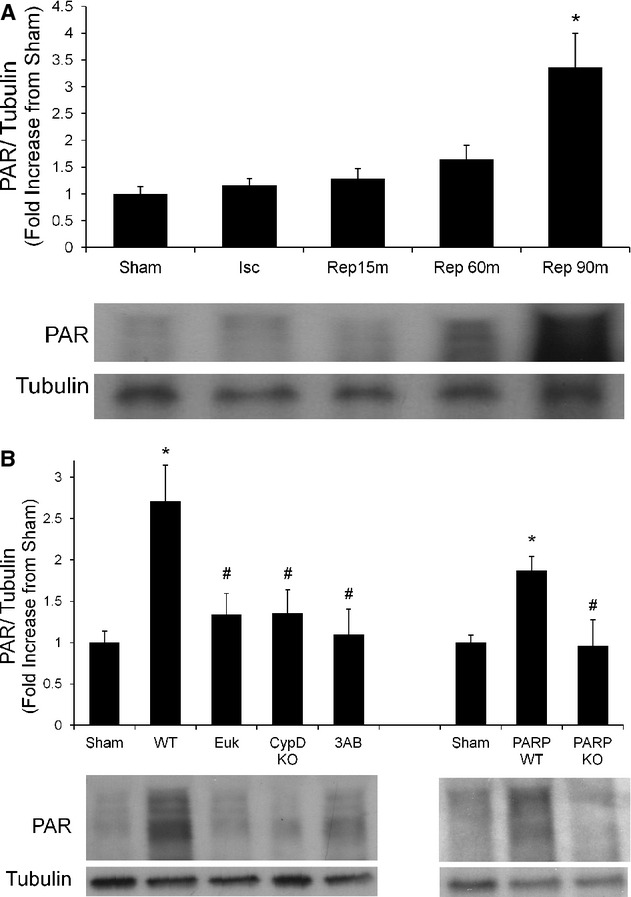
Poly(ADP‐ribosylation) (PAR) after I/R. Immunoblotting of total cell protein from AAR of hearts. A, Wild‐type (WT) hearts subjected to sham occlusion, ischemia (30 minutes) without reperfusion, or ischemia (30 minutes) followed by 15, 60, or 90 minutes of reperfusion, n=5 all groups. B, WT, EUK, CypD‐KO, 3AB, and PARP‐KO hearts after ischemia (30 minutes) and 90 minutes of reperfusion: sham, n=12; WT, n=11; EUK, n=9; CypD, n=10; 3AB, n=10; PARP WT, n=6; PARP KO, n=6. Gel bands were analyzed by densitometry and normalized to tubulin as a loading control. Total PAR protein levels are expressed relative to sham levels. *P<0.05 compared with sham and # compared with WT controls. Values are means±SEMs. I/R indicates ischemia–reperfusion; AAR, area at risk; EUK, EUK134, SODII, and catalase mimetic; CypD, cyclophilin D; 3AB, 3‐aminobenzamide; PARP, poly(ADP‐ribose) polymerase; KO, knockout.

In vitro experiments suggest that PARP activity may be a cause and/or a consequence of ROS or mPTP opening.^35,37,44–45^ Conceivably, PARP activation during I/R could lead to poly(ADP‐ribosylation) of proteins involved in ROS production or mPTP opening, thereby contributing to cell death. If so, then poly(ADP‐ribosylation) of proteins should be evident before the reperfusion burst of ROS and mPTP opening, as these events occur within 15 minutes of reperfusion.^14,19,46^ We observed that poly(ADP‐ribosylated) proteins could only be detected in the AAR of wild‐type hearts at 90 minutes of reperfusion (Figure 3A), which is later than reperfusion ROS or mPTP opening occurs.^14,19,46^ We then tested whether oxidant stress or mPTP opening is required for PARP activity by blotting for poly(ADP‐ribosylated) proteins in hearts treated with PARP inhibitors or antioxidants or lacking CypD or PARP‐1 at 90 minutes of reperfusion. EUK, CypD KO, 3AB, and PARP‐1 KO all prevented the accumulation of total poly(ADP‐ribosylated) proteins (Figure 3B). This indicates that oxidant stress and mPTP opening are both required for PARP‐mediated modification of proteins after I/R.

### Depletion of Cellular NAD(H) in I/R Requires Oxidant Stress, mPTP Opening, and PARP Activity

**Figure 4.**
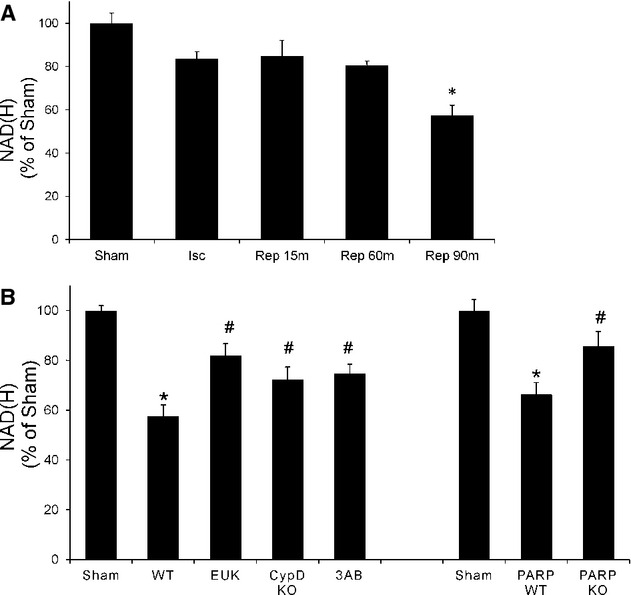
NAD(H) depletion after I/R. NAD(H) levels in the AAR. A, Wild‐type (WT) hearts subjected to sham occlusion, ischemia (30 minutes) without reperfusion, or ischemia (30 minutes) followed by 15, 60, or 90 minutes of reperfusion, n=5 all groups. B, WT, EUK, CypD‐KO, 3AB, and PARP‐KO hearts after 30 minutes of ischemia and 90 minutes of reperfusion: sham, n=6; WT, n=9; EUK, n=7; CypD, n=7; 3AB, n=8; PARP WT, n=6; PARP KO, n=6. NADH levels were normalized to total protein concentration and then normalized to sham levels. *P<0.05 compared with sham and # compared with WT controls. Values are means±SEMs. I/R indicates ischemia–reperfusion; AAR, area at risk; EUK, EUK134, SODII, and catalase mimetic; CypD, cyclophilin D; 3AB, 3‐aminobenzamide; PARP, poly(ADP‐ribose) polymerase; KO, knockout.

Excessive activation of PARP can lead to NAD(H) depletion, thereby inducing a bioenergetic crisis.^31^ In wild‐type hearts subjected to I/R, a progressive loss of NAD(H) during reperfusion was detected, which became significant by 90 minutes (Figure 4A). This paralleled the increase in protein poly(ADP‐ribosylation) as a measure of PARP activity. To assess the contributions of oxidant stress, mPTP, and PARP activity to NAD(H) depletion, we measured the loss of total cellular NAD(H) at 90 minutes of reperfusion. EUK, CypD KO, 3AB, and PARP‐1 KO all maintained higher levels of cellular NAD(H) than did wild‐type controls at 90 minutes of reperfusion (Figure 4B). This indicates that PARP‐mediated consumption of NAD^+^ is attenuated in EUK and CypD‐KO samples, as well as in PARP‐inhibited animals. These findings provide evidence that both oxidant stress and mPTP opening are required for PARP‐mediated NAD^+^ depletion. These results are also consistent with previous work showing that cyclosporin A prevents depletion of total NAD^+^ in response to I/R or excessive DNA damage.^16,44^

### Redistribution of Mitochondrial NAD(H) in I/R Requires Oxidant Stress and mPTP Opening, But Does Not Require PARP Activity

**Figure 5.**
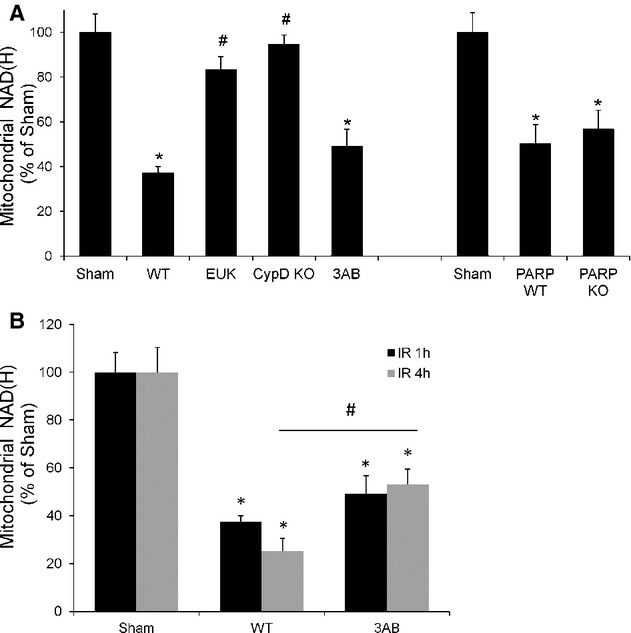
Mitochondrial NAD(H) after I/R. A, NAD(H) in mitochondrial isolates following ischemia (30 minutes) followed by 60 minutes of reperfusion: sham, n=7; WT, n=6; EUK, n=6; CypD, n=7; 3AB, n=8; PARP WT, n=10; PARP KO, n=10. Values are expressed relative to sham group. B, NAD(H) in mitochondrial fractions assayed after 1 and 4 hours of reperfusion, n=6 all groups. Values are expressed relative to sham group. *P<0.05 compared with sham and # compared with WT controls. Values are means±SEMs. I/R indicates ischemia–reperfusion; AAR, area at risk; EUK, EUK134, SODII, and catalase mimetic; CypD, cyclophilin D; 3AB, 3‐aminobenzamide; PARP, poly(ADP‐ribose) polymerase; KO, knockout.

In the heart, most of the cellular NAD(H) is localized to the mitochondria.^47^ Opening of the mPTP causes depletion of mitochondrial NAD(H).^16^ We next asked whether oxidant stress or PARP activity is required for mPTP opening by assaying for loss of mitochondrial NAD(H) after I/R. Mitochondrial NAD(H) content in wild‐type hearts decreased to 37% of sham hearts by 60 minutes of reperfusion. EUK or CypD KO largely prevented this loss, indicating that mPTP opening requires oxidant stress and CypD. By contrast, 3AB and PARP‐1 KO hearts still exhibited significant decreases in mitochondrial NAD(H), suggesting that the mPTP had opened in these hearts (Figure 5A). These findings indicate that ROS is required to induce mPTP opening, whereas PARP activity is not. These results place oxidant stress upstream and PARP activity downstream of mPTP‐mediated loss of mitochondrial NAD(H).

### PARP Activity Contributes to Further Mitochondrial NAD(H) Loss During Reperfusion

If PARP activity is downstream of mPTP opening, then PARP inhibition could protect by either allowing mitochondria to recover from initial mPTP opening or by preventing further mitochondrial dysfunction. To test this, we assayed for recovery of mitochondrial NAD(H) levels during reperfusion. One hour into reperfusion, mitochondrial NAD(H) levels were similar in wild‐type and 3AB‐treated hearts, but by 4 hours of reperfusion NAD(H) levels were significantly greater in 3AB hearts than in WT hearts (Figure 5B). These findings suggest that PARP inhibition prevents further loss of mitochondrial NAD(H) after the initial mPTP‐mediated loss occurs.

### Loss of Cellular ATP at 1.5 Hours of Reperfusion Does Not Require PARP Activity, Whereas Sustained Depletion of ATP by 4 Hours of Reperfusion Requires PARP Activity

**Figure 6.**
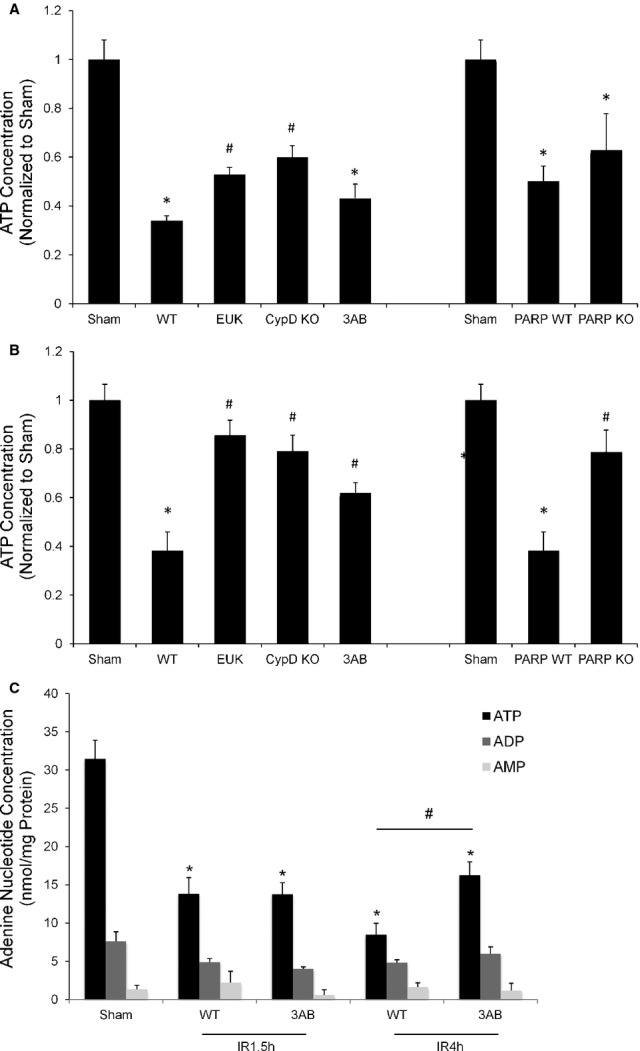
ATP and total adenine nucleotide levels after I/R. Total ATP levels in the AAR of hearts following ischemia (30 minutes) followed by (A) 1.5 hours or (B) 4 hours of reperfusion. C, Total adenine nucleotide levels in the AAR of hearts following ischemia (30 minutes) followed by 4 hours of reperfusion. At 1.5 hours, sham, n=6; WT, n=5; EUK, n=6; CypD, n=6; 3AB, n=6; PARP WT, n=6; PARP KO, n=6. At 4 hours, sham, n=5; WT, n=7; EUK, n=6; CypD, n=6; 3AB, n=6; PARP WT, n=6; PARP KO, n=6. Total adenine nucleotide levels, sham, n=8; WT, n=8; 3AB, n=8. Values are expressed relative to sham group. *P<0.05 compared with sham and # compared with WT controls. Values are means±SEMs. I/R indicates ischemia–reperfusion; AAR, area at risk; WT, wild type; EUK, EUK134, SODII, and catalase mimetic; CypD, cyclophilin D; 3AB, 3‐aminobenzamide; PARP, poly(ADP‐ribose) polymerase; KO, knockout.

Opening of the mPTP causes mitochondrial depolarization, which abolishes ATP generation by oxidative phosphorylation and may further enhance ATP depletion via reverse operation of the FoF1 ATP synthase.^25^ To investigate the role of PARP activity in ATP depletion after I/R, we measured ATP levels in the AAR of reperfused hearts. The 3AB‐treated and PARP‐1‐KO hearts showed similar losses of ATP at 1.5 hours of reperfusion compared with their wild‐type controls. However, EUK‐treated and CypD‐KO hearts had significantly greater ATP levels (Figure 6A). These results indicate that early mPTP‐mediated depletion of total ATP does not require PARP activity, which strengthens the argument that PARP‐mediated damage occurs downstream of mPTP opening. By 4 hours of reperfusion, however, PARP‐inhibited samples as well as EUK‐treated and CypD‐KO hearts all exhibited significantly greater ATP levels compared with WT hearts (Figure 6B). To assess whether PARP enhances adenine nucleotide loss or prevents ATP recovery, beating hearts were rapidly freeze‐clamped in situ to obtain accurate assessments of total adenine nucleotide levels in the area at risk. After 1.5 hours of reperfusion, total adenine nucleotide levels were significantly suppressed in both wild‐type and PARP‐inhibited hearts compared with shams. After 4 hours of reperfusion the adenine nucleotides in 3AB‐treated hearts were not decreased from the 1.5‐hour levels, whereas WT hearts showed a further decrease (Figure 6C). This indicates that PARP activity during reperfusion is responsible for further ATP depletion after the initial mPTP‐mediated ATP depletion has occurred.

### Mitochondria Progressively Depolarize Throughout Reperfusion Before Plasma Membrane Rupture

**Figure 7.**
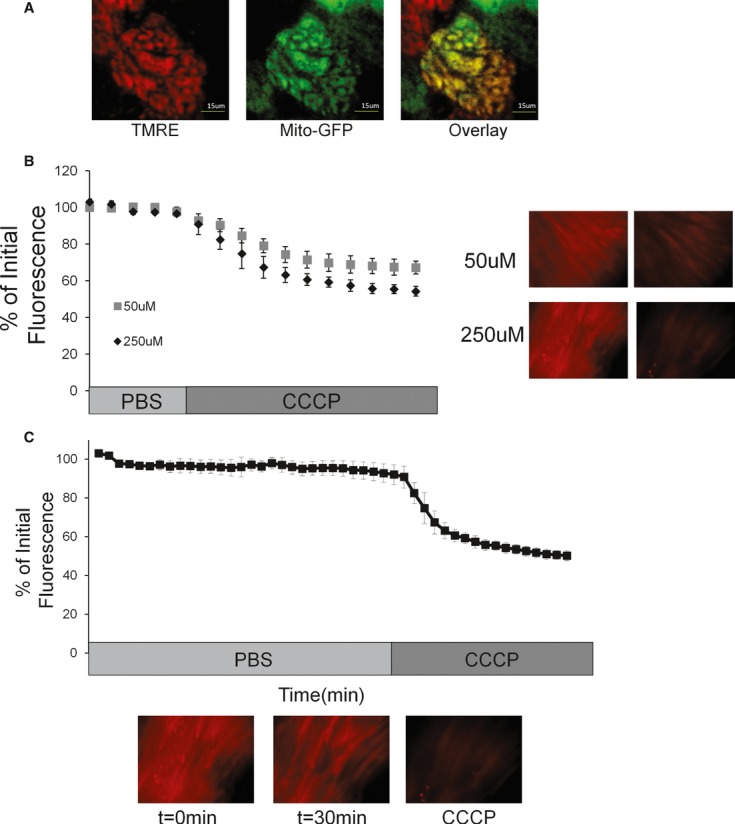
TMRE localization, stability, and responses to CCCP *ex vivo*. A, Mitochondrial‐targeted GFP‐expressing hearts were loaded with TMRE in vivo, excised, and sliced. Colocalization of GFP and TMRE fluorescence was confirmed by 2‐photon microscopy. B, Hearts were loaded with TMRE, excised, sliced, and imaged during CCCP (50 or 250 μmol/L) treatment for 15 minutes. C, Hearts were loaded with TMRE, excised, sliced, and imaged while being maintained in ice‐cold PBS for 30 minutes followed by 15 minutes of treatment with 250 μmol/L CCCP, n=5 all groups. TMRE indicates tetramethylrodamine ethyl ester; CCCP, carbonyl cyanide *m*‐chlorophenyl hydrazone; GFP, green fluorescent protein; PBS, phosphate‐buffered saline.

**Figure 8.**
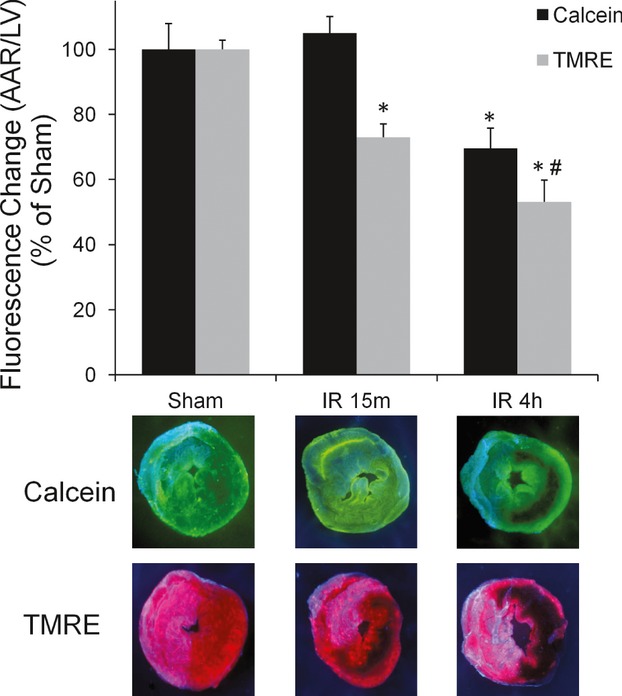
Mitochondrial polarization in relation to plasma membrane rupture after I/R. TMRE fluorescence and calcein‐AM staining at different times after reperfusion. TMRE was loaded into beating hearts for 15 minutes. TMRE and calcein fluorescence change in hearts subjected to ischemia (30 minutes) followed by reperfusion for 15 minutes or 4 hours, n=5 all groups. *P<0.05 compared with sham and # compared with 15‐minute time. Values are means±SEMs. I/R indicates ischemia–reperfusion; TMRE, tetramethylrodamine ethyl ester; AAR, area at risk; LV, left ventricle.

To assess the effects of PARP activity on reperfusion‐induced depolarization, we developed an in vivo assay of mitochondrial potential using the potentiometric dye tetramethylrhodamine ethyl ester (TMRE). This probe distributes across membranes in accordance with the Nernst potential and has been used extensively to study mitochondrial potential, even in the intact heart.^48–49^ To test whether TMRE fluorescence intensity in tissue is exclusively mitochondrial, hearts constitutively expressing mitochondrial‐targeted green fluorescent protein (GFP) were loaded with TMRE and imaged. TMRE localizes exclusively in the mitochondria (Figure 7A). To test whether hearts loaded with TMRE are responsive to a mitochondrial uncoupler, hearts were loaded with TMRE in vivo, excised, sliced, and treated with the uncoupler carbonyl cyanide *m*‐chlorophenyl hydrazone (CCCP; 50 and 250 μmol/L). Significant loss of fluorescence was observed in CCCP‐treated samples after 15 minutes of incubation compared with the first 5 minutes of perfusion with PBS, indicating that TMRE fluorescence decreases when mitochondria are depolarized (Figure 7B). To assess the stability of TMRE fluorescence in ex vivo heart slices, hearts were loaded, excised and sliced, and maintained in ice‐cold PBS on a fluorescence imaging system. Slices maintained in cold PBS showed no decrease in fluorescence over 30 minutes, indicating that TMRE fluorescence is stable over time (Figure 7C). Using this approach we then assessed mitochondrial polarization after I/R. TMRE was administered to mice immediately after reperfusion and allowed to equilibrate for 15 minutes. Hearts were then removed, sliced, and evaluated. Hearts loaded with TMRE and studied 15 minutes after reperfusion show significantly less fluorescence in the area at risk than sham hearts. However, the cells were still able to accumulate calcein‐AM dye, indicating that the plasma membrane was then still intact.^50^ Hence, the lack of TMRE fluorescence in the AAR at 15 minutes of reperfusion was not a consequence of tissue necrosis. By contrast, after 4 hours of reperfusion, both TMRE and calcein‐AM staining in the AAR was significantly lower than in sham hearts (Figure 8). These studies show that TMRE accumulates in polarized mitochondria in the intact heart, that loss of mitochondrial potential caused by I/R decreases TMRE staining, and that absence of TMRE staining can be seen in depolarized mitochondria before cells have died.

### PARP Activity Causes Further Loss of Mitochondrial Potential During 4 Hours of Reperfusion

**Figure 9.**
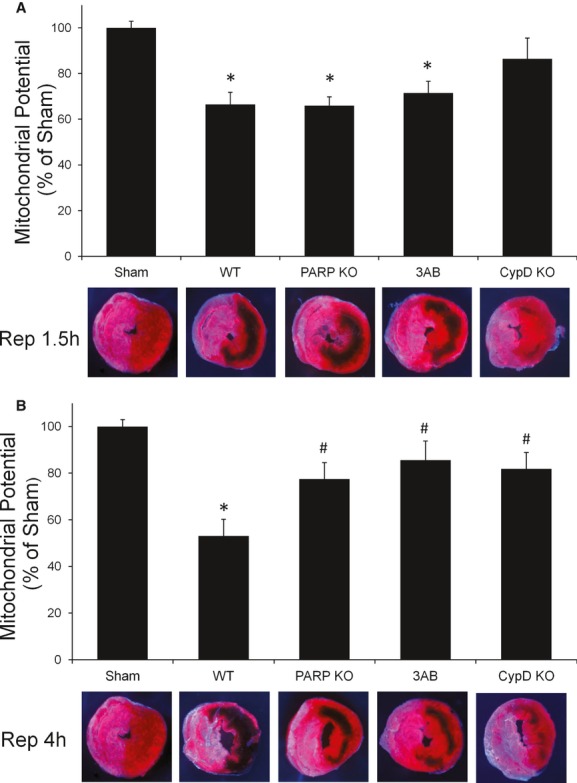
Mitochondrial polarization after I/R. Hearts were loaded with TMRE at different points after reperfusion. Hearts were then retroperfused with Hoechst to delineate the AAR, sliced, and imaged. Total fluorescence of the AAR was compared with the area not at risk and normalized to sham hearts. Fluorescence changes from genetically matched WT, 3AB, PARP‐1‐KO, and CypD‐KO hearts were compared after (A) 1.5 hours or (B) 4 hours of reperfusion. Included are representative heart slices showing TMRE voids in the AAR, n=5 all groups. *P<0.05 compared with sham and # compared with WT controls. Values are means±SEMs. I/R indicates ischemia–reperfusion; TMRE, tetramethylrodamine ethyl ester; AAR, area at risk; WT, wild type; CypD, cyclophilin D; 3AB, 3‐aminobenzamide; PARP, poly(ADP‐ribose) polymerase; KO, knockout.

We then sought to determine whether PARP activity contributes to a decline in mitochondrial potential over time during reperfusion. First, we assayed for mitochondrial potential at 1.5 hours of reperfusion. Fluorescence loss was evident in PARP‐1‐KO, PARP‐inhibited, and WT hearts, suggesting that mPTP‐mediated depolarization had occurred. By contrast, CypD‐KO hearts showed less decline at 1.5 hours, consistent with inhibition of mPTP opening (Figure 9A). We then assayed for mitochondrial potential at 4 hours of reperfusion. By 4 hours, fluorescence was significantly greater in PARP‐1‐KO, PARP‐inhibited, and CypD hearts compared with WT hearts. Importantly, fluorescence levels in PARP‐1‐KO and PARP‐inhibited hearts were no longer statistically different from sham hearts (Figure 9B). These findings indicate that CypD contributes to the transient loss of mitochondrial potential early in reperfusion and that subsequent activation of PARP contributes to further loss of mitochondrial bioenergetic function during the later stages of reperfusion.

### Although Plasma Membrane Rupture Is Evident at 4 Hours of Reperfusion, PARP‐Mediated Decline in Mitochondrial Function During 4 Hours of Reperfusion Is Not Caused by Release of Proapoptotic Proteins to the Cytosol

**Figure 10.**
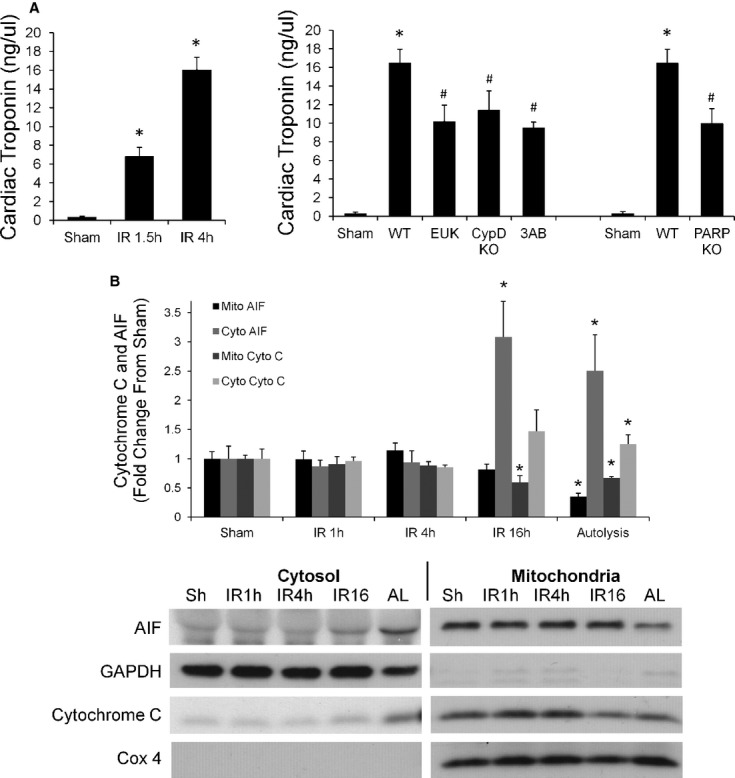
Cardiac troponin release in relation to cytochrome c or AIF translocation after I/R. A, Cardiac troponin in circulating plasma as a measure of plasma membrane rupture after 1.5 and 4 hours of reperfusion and in EUK, CypD‐KO, 3AB, and PARP‐KO hearts at 4 hours of reperfusion. B, Mitochondrial AIF, mitochondrial cytochrome c, and cytosolic AIF and cytochrome c in cellular fractions in sham hearts or after ischemia (30 minutes) followed by reperfusion for 1 hour (IR1h), 4 hours (IR4h), 16 hours (IR16h), or *ex vivo* hearts allowed to autolyse for 1 hour (AL): sham, n=6; IR1.5h, n=5; IR4h, n=6; IR16h, n=6; AL, n=5. Mitochondrial values are normalized to cytochrome oxidase subunit 4 (Cox IV), and cytosolic values are normalized to GAPDH. *P<0.05 compared with sham. Values are means±SEMs. AIF indicates apoptosis‐inducing factor; I/R, ischemia–reperfusion; AAR, area at risk; WT, wild type; EUK, EUK134, SODII, and catalase mimetic; CypD, cyclophilin D; 3AB, 3‐aminobenzamide; PARP, poly(ADP‐ribose) polymerase; KO, knockout; Cox IV, cytochrome c oxidase subunit 4.

The interdependence of outer mitochondrial membrane rupture and plasma membrane rupture after I/R has not been explored. As TTC staining heavily relies on mitochondrial function to indicate necrosis, cardiac troponin release, a more direct measure of plasma membrane rupture, was measured. Cardiac troponin progressively accumulates in the bloodstream after reperfusion, indicating loss of sarcolemmal integrity (Figure 10A). Antioxidants, CypD KO, and PARP inhibition all significantly blunt cardiac troponin release at 4 hours of reperfusion, indicating that they preserve cellular integrity. PARP activity has been reported to induce translocation of proapoptotic proteins to the cytosol after an acute necrotic stimulus.^51^ The mechanism of PARP‐mediated loss of mitochondrial function we observed could conceivably result from mitochondrial outer membrane rupture subsequent to mPTP opening but before plasma membrane rupture. To test this, we assessed mitochondrial and cytosolic levels of cytochrome c and AIF by immunoblotting. After 1 and 4 hours of reperfusion, mitochondrial and cytosolic levels of cytochrome c and AIF were unchanged in wild‐type I/R samples, whereas redistribution was evident by 16 hours into reperfusion or in *ex vivo* hearts maintained at 37°C for 1 hour (Figure 10B). This indicates that although plasma membrane rupture is evident at 4 hours of reperfusion, outer mitochondrial membrane rupture has not yet occurred. Therefore, PARP‐mediated mitochondrial dysfunction is not caused by rupture of the outer mitochondrial membrane, but rather appears to arise from a bioenergetic crisis arising from sustained mitochondrial depolarization.

## Discussion

**Figure 11.**
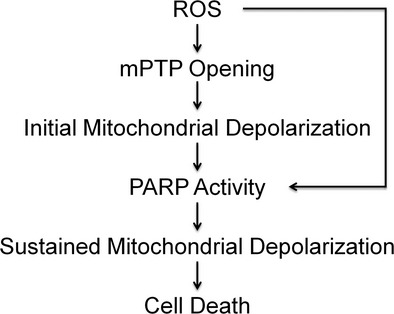
Model of myocardial I/R‐induced cell death. I/R indicates ischemia–reperfusion; ROS, reactive oxygen species; mPTP, mitochondrial permeability transition pore; PARP, poly(ADP‐ribose) polymerase.

This study links 3 known yet distinct death‐inducing events into an integrated cell death pathway induced by I/R in the intact, blood‐perfused heart (Figure 11). First, oxidant stress has long been linked to reperfusion‐induced cell death in the heart, although the question of whether oxidant stress is directly lethal or instead represents a death signal is not fully known.^52^ Previous studies have also suggested that oxidant stress is linked to activation of the mPTP, which has primarily represented an irreversible and lethal event arising from mitochondrial matrix swelling followed by rupture of the outer membrane.^21,53^ Accordingly, inhibition of the mPTP—by genetic deletion of the gene encoding CypD or by treatment with cyclosporin A—confers protection against reperfusion‐induced cell death.^6,16^ However, other studies have shown that PARP inactivation confers protection to a similar extent.^7,30^ If these observations are correct, then either (1) PARP activation contributes to cell death by modifying mPTP opening, either directly or through differential oxidant generation, or (2) PARP and mPTP opening trigger death in parallel independent pathways, possibly by affecting different cell populations. Therefore, the present study sought to clarify the relationship between oxidant stress, PARP, and mPTP‐mediated mechanisms of death in the blood‐perfused, LAD I/R model.

One way in which PARP and the mPTP could interact is through differential generation of oxidant stress. For example, mPTP opening could generate oxidant stress that causes DNA damage and activates PARP, or PARP activity could interfere with mitochondrial function, causing oxidant generation that could open the mPTP. Our data show that significant oxidative damage to reperfused tissue is required to induce mPTP opening and PARP activation and that oxidative stress and mPTP opening precede PARP activation. These findings place oxidant stress as the proximal trigger of both events, suggesting that the potential interaction between the mPTP and PARP is downstream of reperfusion‐induced oxidant generation.

A second potential mechanism of interaction could involve PARP directly causing mPTP opening at reperfusion. However, our data show that neither the decrease in mitochondrial potential nor the decrease in mitochondrial NAD(H) after 1 to 1.5 hours of reperfusion was prevented by PARP deletion or inhibition; thus, PARP activity is not required for mPTP activation. Moreover, PARP inhibition did not prevent oxidative damage or early mPTP‐mediated loss of mitochondrial function, indicating that ROS generation and mPTP activation are PARP‐independent events in early reperfusion.

A third mechanism of cell death could involve PARP and the mPTP acting in separate pathways, possibly in distinct cell populations. However, hearts lacking CypD or PARP‐1 exhibited similar levels of cell death after 4 hours of reperfusion, as seen by TTC staining and cardiac troponin release, and inhibition of PARP in CypD‐KO hearts did not confer significant additional protection. These observations suggest that the mPTP and PARP systems may act in the same linear cell death pathway.

If PARP activity regulates cell death subsequent to mPTP opening, this would imply that mPTP activation is not immediately lethal to the cell. So how does PARP activity contribute to cell death after the mPTP has opened? On reperfusion, the mitochondria undergo an initial mPTP‐dependent depolarization. After between 1.5 and 4 hours we observed a further decline in mitochondrial membrane potential, NAD(H) concentration, and ATP levels in wild‐type animals. By contrast, PARP‐inhibited animals showed no further decline in mitochondrial NAD(H), ATP concentration, or mitochondrial potential after the 1.5‐hour point. These findings indicate that PARP activity undermines mitochondrial function and/or recovery during this intermediate period of reperfusion. One possible mechanism is that PARP activation inhibits the restoration of ATP, ADP, and AMP by inhibiting the adenine nucleotide salvage pathway. However, this mechanism does not explain why mitochondrial potential was better preserved in the PARP‐KO and PARP‐inhibited hearts between the 1.5‐ and 4‐hour points, as mitochondrial polarization is not dependent on ATP levels once tissue oxygen levels are restored.

An alternative explanation is that some mitochondria can reclose the mPTP and repolarize during reperfusion if PARP activity is prevented. In this scenario, the initial ROS generation and mPTP opening at reperfusion trigger PARP activity, which then contributes to a further decline in mitochondrial function after between 1.5 and 4 hours of reperfusion. Consistent with this model, hearts lacking PARP activity tended to recover mitochondrial membrane potential and ATP levels during this period, whereas the control hearts exhibited further decline. These observations suggest that PARP contributes to cell death by inhibiting the recovery of mitochondria that have undergone permeability transition at the start of reperfusion. This provocative interpretation contradicts the canonical model in which mPTP opening commits a cell to death via rapid and irreversible matrix swelling and release of prodeath proteins from the mitochondrial intermembrane space.

The idea that mPTP activation irreversibly commits a cell to a death pathway is based on the in vitro observation that mPTP opening causes sustained depolarization followed by mitochondrial matrix swelling and outer membrane rupture.^53^ However, nonlethal mPTP‐mediated depolarization has been reported,^17,54–55^ and sublethal ischemia results in mPTP‐mediated depolarization followed by repolarization at reperfusion.^54^ Other work suggested that the mPTP can close after reperfusion and that this is augmented by high‐energy substrates such as pyruvate.^55^ Previous studies of reperfused myocardium reported that apoptotic pathways peak 24 to 72 hours after reperfusion and that AIF and cytochrome c are released from the mitochondria in a PARP‐dependent manner after 6 to 12 hours of reperfusion.^56–57^ In addition, a careful study of mitochondrial outer membrane rupture as the result of mPTP opening showed that in vitro swelling assays promote massive mitochondrial membrane rupture, whereas in vivo I/R‐mediated outer mitochondrial membrane rupture is much less severe.^58^ In our study, no evidence of AIF or cytochrome c release was detected in the AAR by 4 hours of reperfusion despite release of cardiac troponin and loss of TTC staining, whereas AIF and cytochrome c release were seen at 16 hours. These findings suggest that activation of the mPTP in this model of I/R is not associated with immediate swelling and rupture. Rather, I/R‐mediated cell death occurs as a consequence of prolonged mitochondrial depolarization caused by PARP‐mediated energy failure. Thus, PARP activity undermines the recovery of mitochondrial potential throughout reperfusion, permitting later release of mitochondrial prodeath factors as a secondary consequence of the failure to recover mitochondrial polarization after mPTP opening.

PARP activity has been suggested to undermine mitochondrial function by causing a bioenergetic crisis; however, the precise mechanism is unknown.^47^ In the present study we have described a new model by which PARP can affect mitochondrial function by inhibiting ATP synthesis downstream of mPTP opening. During reperfusion, closure of the mPTP and rescue of the cell might be facilitated by mitochondrial repolarization achieved using glycolytic ATP to drive ATP synthase in reverse, thus reestablishing a proton gradient. PARP activity could undermine this recovery by depleting cytosolic NAD^+^, thereby limiting the ability to generate ATP by glycolysis. NAD^+^ depletion could also undermine the recovery of ATP generation, as the de novo synthesis of NAD^+^—which is required for glycolysis—requires ATP availability. Finally, PARP activity could directly inhibit transport of adenine nucleotides into the mitochondria,^59^ thereby preventing cytosolic ATP from reaching the matrix, where it could facilitate repolarization. This cycle of ATP depletion would cause sustained depolarization, leading to mitochondrial failure and plasma membrane rupture without affecting outer mitochondrial membrane integrity.

In accordance with this model, PARP inhibitors have been shown to protect the myocardium and the brain even when administered up to 4 hours after reperfusion when infarct size was assessed at 24 hours.^60–61^ Our data therefore explain how PARP inhibition provides protection downstream of mPTP opening by revealing that mPTP‐mediated depolarization is not terminal in a significant fraction of the cells in the area at risk. From a therapeutic standpoint, these findings suggest that attenuation of myocardial I/R injury could be achieved by limiting PARP activity even though the initial mPTP opening may have already occurred.

## References

[1] Roger VL Go AS Lloyd‐Jones DM Benjamin EJ Berry JD Borden WB Bravata DM Dai S Ford ES Fox CS Fullerton HJ Gillespie C Hailpern SM Heit JA Howard VJ Kissela BM Kittner SJ Lackland DT Lichtman JH Lisabeth LD Makuc DM Marcus GM Marelli A Matchar DB Moy CS Mozaffarian D Mussolino ME Nichol G Paynter NP Soliman EZ Sorlie PD Sotoodehnia N Turan TN Virani SS Wong ND Woo D Turner MB Heart disease and stroke statistics—2012 update: a report from the American Heart Association. Circulation. 2012; 125:e2-e220 22179539 10.1161/CIR.0b013e31823ac046 PMC4440543

[2] Yellon DM Hausenloy DJ Myocardial reperfusion injury. N Engl J Med. 2007; 357:1121-1135 17855673 10.1056/NEJMra071667

[3] Powers SK Murlasits Z Wu M Kavazis AN Ischemia–reperfusion‐induced cardiac injury: a brief review. Med Sci Sports Exerc. 2007; 39:1529-1536 17805085 10.1249/mss.0b013e3180d099c1

[4] Levraut J Iwase H Shao ZH Vanden Hoek TL Schumacker PT Cell death during ischemia: relationship to mitochondrial depolarization and ROS generation. Am J Physiol Heart Circ Physiol. 2003; 284:H549-H558 12388276 10.1152/ajpheart.00708.2002

[5] Chen Z Siu B Ho YS Vincent R Chua CC Hamdy RC Chua BH Overexpression of MnSOD protects against myocardial ischemia/reperfusion injury in transgenic mice. J Mol Cell Cardiol. 1998; 30:2281-2289 9925365 10.1006/jmcc.1998.0789

[6] Baines CP Kaiser RA Purcell NH Blair NS Osinska H Hambleton MA Brunskill EW Sayen MR Gottlieb RA Dorn GW Robbins J Molkentin JD Loss of cyclophilin D reveals a critical role for mitochondrial permeability transition in cell death. Nature. 2005; 434:658-662 15800627 10.1038/nature03434

[7] Yang Z Zingarelli B Szabo C Effect of genetic disruption of poly (ADP‐ribose) synthetase on delayed production of inflammatory mediators and delayed necrosis during myocardial ischemia–reperfusion injury. Shock. 2000; 13:60-66 10638671 10.1097/00024382-200013010-00011

[8] Loor G Kondapalli J Iwase H Chandel NS Waypa GB Guzy RD Vanden Hoek TL Schumacker PT Mitochondrial oxidant stress triggers cell death in simulated ischemia–reperfusion. Biochim Biophys Acta. 2011; 1813:1382-1394 21185334 10.1016/j.bbamcr.2010.12.008 PMC3089816

[9] Kowaltowski AJ Vercesi AE Mitochondrial damage induced by conditions of oxidative stress. Free Radic Biol Med. 1999; 26:463-471 9895239 10.1016/s0891-5849(98)00216-0

[10] Robin E Guzy RD Loor G Iwase H Waypa GB Marks JD Hoek TL Schumacker PT Oxidant stress during simulated ischemia primes cardiomyocytes for cell death during reperfusion. J Biol Chem. 2007; 282:19133-19143 17488710 10.1074/jbc.M701917200

[11] Grill HP Zweier JL Kuppusamy P Weisfeldt ML Flaherty JT Direct measurement of myocardial free radical generation in an in vivo model: effects of postischemic reperfusion and treatment with human recombinant superoxide dismutase. J Am Coll Cardiol. 1992; 20:1604-1611 1333498 10.1016/0735-1097(92)90457-x

[12] Xu Y Liu B Zweier JL He G Formation of hydrogen peroxide and reduction of peroxynitrite via dismutation of superoxide at reperfusion enhances myocardial blood flow and oxygen consumption in postischemic mouse heart. J Pharmacol Exp Ther. 2008; 327:402-410 18685120 10.1124/jpet.108.142372 PMC2615247

[13] Jolly SR Kane WJ Bailie MB Abrams GD Lucchesi BR Canine myocardial reperfusion injury. Its reduction by the combined administration of superoxide dismutase and catalase. Circ Res. 1984; 54:277-285 6697450 10.1161/01.res.54.3.277

[14] Halestrap AP Connern CP Griffiths EJ Kerr PM Cyclosporin A binding to mitochondrial cyclophilin inhibits the permeability transition pore and protects hearts from ischaemia/reperfusion injury. Mol Cell Biochem. 1997; 174:167-172 9309682

[15] Lemasters JJ Qian T Bradham CA Brenner DA Cascio WE Trost LC Nishimura Y Nieminen AL Herman B Mitochondrial dysfunction in the pathogenesis of necrotic and apoptotic cell death. J Bioenerg Biomembr. 1999; 31:305-319 10665521 10.1023/a:1005419617371

[16] Di Lisa F Menabo R Canton M Barile M Bernardi P Opening of the mitochondrial permeability transition pore causes depletion of mitochondrial and cytosolic NAD+ and is a causative event in the death of myocytes in postischemic reperfusion of the heart. J Biol Chem. 2001; 276:2571-2575 11073947 10.1074/jbc.M006825200

[17] Ichas F Mazat JP From calcium signaling to cell death: two conformations for the mitochondrial permeability transition pore. Switching from low‐ to high‐conductance state. Biochim Biophys Acta. 1998; 1366:33-50 9714722 10.1016/s0005-2728(98)00119-4

[18] Petronilli V Costantini P Scorrano L Colonna R Passamonti S Bernardi P The voltage sensor of the mitochondrial permeability transition pore is tuned by the oxidation‐reduction state of vicinal thiols. Increase of the gating potential by oxidants and its reversal by reducing agents. J Biol Chem. 1994; 269:16638-16642 7515881

[19] Kim JS Jin Y Lemasters JJ Reactive oxygen species, but not Ca^2+^ overloading, trigger pH‐ and mitochondrial permeability transition‐dependent death of adult rat myocytes after ischemia‐reperfusion. Am J Physiol Heart Circ Physiol. 2006; 290:H2024-H2034 16399872 10.1152/ajpheart.00683.2005

[20] Assaly R de Tassigny A Paradis S Jacquin S Berdeaux A Morin D Oxidative stress, mitochondrial permeability transition pore opening and cell death during hypoxia‐reoxygenation in adult cardiomyocytes. Eur J Pharmacol. 2012; 675:6-14 22173126 10.1016/j.ejphar.2011.11.036

[21] Clarke SJ Khaliulin I Das M Parker JE Heesom KJ Halestrap AP Inhibition of mitochondrial permeability transition pore opening by ischemic preconditioning is probably mediated by reduction of oxidative stress rather than mitochondrial protein phosphorylation. Circ Res. 2008; 102:1082-1090 18356542 10.1161/CIRCRESAHA.107.167072 PMC2629616

[22] Zorov DB Filburn CR Klotz LO Zweier JL Sollott SJ Reactive oxygen species (ROS)‐induced ROS release: a new phenomenon accompanying induction of the mitochondrial permeability transition in cardiac myocytes. J Exp Med. 2000; 192:1001-1014 11015441 10.1084/jem.192.7.1001 PMC2193314

[23] Nakagawa T Shimizu S Watanabe T Yamaguchi O Otsu K Yamagata H Inohara H Kubo T Tsujimoto Y Cyclophilin D‐dependent mitochondrial permeability transition regulates some necrotic but not apoptotic cell death. Nature. 2005; 434:652-658 15800626 10.1038/nature03317

[24] Schinzel AC Takeuchi O Huang Z Fisher JK Zhou Z Rubens J Hetz C Danial NN Moskowitz MA Korsmeyer SJ Cyclophilin D is a component of mitochondrial permeability transition and mediates neuronal cell death after focal cerebral ischemia. Proc Natl Acad Sci USA. 2005; 102:12005-12010 16103352 10.1073/pnas.0505294102 PMC1189333

[25] Abdallah Y Iraqi W Said M Kasseckert SA Shahzad T Erdogan A Neuhof C Gunduz D Schluter KD Piper HM Reusch HP Ladilov Y Interplay between Ca(2+) cycling and mitochondrial permeability transition pores promotes reperfusion‐induced injury of cardiac myocytes. J Cell Mol Med. 2011; 15:2478-2485 21199327 10.1111/j.1582-4934.2010.01249.x PMC3822958

[26] Liaudet L Yang Z Al‐Affar EB Szabo C Myocardial ischemic preconditioning in rodents is dependent on poly (ADP‐ribose) synthetase. Mol Med. 2001; 7:406-417 11474134 PMC1950045

[27] D'Amours D Desnoyers S D'Silva I Poirier GG Poly(ADP‐ribosyl)ation reactions in the regulation of nuclear functions. Biochem J. 1999; 342Pt 2:249-268 10455009 PMC1220459

[28] Virag L Structure and function of poly(ADP‐ribose) polymerase‐1: role in oxidative stress‐related pathologies. Curr Vasc Pharmacol. 2005; 3:209-214 16026317 10.2174/1570161054368625

[29] Berger NA Sims JL Catino DM Berger SJ Poly(ADP‐ribose) polymerase mediates the suicide response to massive DNA damage: studies in normal and DNA‐repair defective cells. Princess Takamatsu Symp. 1983; 13:219-226 6317637

[30] Halmosi R Berente Z Osz E Toth K Literati‐Nagy P Sumegi B Effect of poly(ADP‐ribose) polymerase inhibitors on the ischemia‐reperfusion‐induced oxidative cell damage and mitochondrial metabolism in Langendorff heart perfusion system. Mol Pharmacol. 2001; 59:1497-1505 11353811 10.1124/mol.59.6.1497

[31] Schraufstatter IU Hinshaw DB Hyslop PA Spragg RG Cochrane CG Oxidant injury of cells. DNA strand‐breaks activate polyadenosine diphosphate‐ribose polymerase and lead to depletion of nicotinamide adenine dinucleotide. J Clin Invest. 1986; 77:1312-1320 2937805 10.1172/JCI112436 PMC424485

[32] Yu S‐W Andrabi SA Wang H Kim NS Poirier GG Dawson TM Dawson VL Apoptosis‐inducing factor mediates poly(ADP‐ribose) (PAR) polymer‐induced cell death. Proc Natl Acad Sci USA. 2006; 103:18314-18319 17116881 10.1073/pnas.0606528103 PMC1838748

[33] Yu SW Wang H Poitras MF Coombs C Bowers WJ Federoff HJ Poirier GG Dawson TM Dawson VL Mediation of poly(ADP‐ribose) polymerase‐1‐dependent cell death by apoptosis‐inducing factor. Science. 2002; 297:259-263 12114629 10.1126/science.1072221

[34] Chen M Zsengeller Z Xiao CY Szabo C Mitochondrial‐to‐nuclear translocation of apoptosis‐inducing factor in cardiac myocytes during oxidant stress: potential role of poly(ADP‐ribose) polymerase‐1. Cardiovasc Res. 2004; 63:682-688 15306224 10.1016/j.cardiores.2004.04.018

[35] Zhou HZ Swanson RA Simonis U Ma X Cecchini G Gray MO Poly(ADP‐ribose) polymerase‐1 hyperactivation and impairment of mitochondrial respiratory chain complex I function in reperfused mouse hearts. Am J Physiol Heart Circ Physiol. 2006; 291:H714-H723 16582021 10.1152/ajpheart.00823.2005

[36] Du L Zhang X Han YY Burke NA Kochanek PM Watkins SC Graham SH Carcillo JA Szabo C Clark RSB Intra‐mitochondrial poly(ADP‐ribosylation) contributes to NAD+ depletion and cell death induced by oxidative stress. J Biol Chem. 2003; 278:18426-18433 12626504 10.1074/jbc.M301295200

[37] Alano CC Ying W Swanson RA Poly(ADP‐ribose) polymerase‐1‐mediated cell death in astrocytes requires NAD+ depletion and mitochondrial permeability transition. J Biol Chem. 2004; 279:18895-18902 14960594 10.1074/jbc.M313329200

[38] Xu Y Huang S Liu ZG Han J Poly(ADP‐ribose) polymerase‐1 signaling to mitochondria in necrotic cell death requires RIP1/TRAF2‐mediated JNK1 activation. J Biol Chem. 2006; 281:8788-8795 16446354 10.1074/jbc.M508135200

[39] Michael LH Entman ML Hartley CJ Youker KA Zhu J Hall SR Hawkins HK Berens K Ballantyne CM Myocardial ischemia and reperfusion: a murine model. Am J Physiol. 1995; 269:H2147-H2154 8594926 10.1152/ajpheart.1995.269.6.H2147

[40] Nisselbaum JS Green S A simple ultramicro method for determination of pyridine nucleotides in tissues. Anal Biochem. 1969; 27:212-217 4388021 10.1016/0003-2697(69)90025-6

[41] Santos JBM Van Houten B Measuring oxidative mtDNA damage and repair using quantitative PCR. Methods Mol Biol. 2002; 197:159-176 12013794 10.1385/1-59259-284-8:159

[42] Santos J Meyer JN Mandavilli BS Van Houten B Quantitative PCR‐based measurement of nuclear and mitochondrial DNA damage and repair in mammalian cells. Methods Mol Biol. 2006; 314:183-199 16673882 10.1385/1-59259-973-7:183

[43] Yakes FM Van Houten B Mitochondrial DNA damage is more extensive and persists longer than nuclear DNA damage in human cells following oxidative stress. Proc Natl Acad Sci USA. 1997; 94:514-519 9012815 10.1073/pnas.94.2.514 PMC19544

[44] Alano CC Tran A Tao R Ying W Karliner JS Swanson RA Differences among cell types in NAD(+) compartmentalization: a comparison of neurons, astrocytes, and cardiac myocytes. J Neurosci Res. 2007; 85:3378-3385 17853438 10.1002/jnr.21479

[45] Ying W Sevigny MB Chen Y Swanson RA Poly(ADP‐ribose) glycohydrolase mediates oxidative and excitotoxic neuronal death. Proc Natl Acad Sci USA. 2001; 98:12227-12232 11593040 10.1073/pnas.211202598 PMC59796

[46] Zweier JL Flaherty JT Weisfeldt ML Direct measurement of free radical generation following reperfusion of ischemic myocardium. Proc Natl Acad Sci USA. 1987; 84:1404-1407 3029779 10.1073/pnas.84.5.1404 PMC304438

[47] Alano CC Garnier P Ying W Higashi Y Kauppinen TM Swanson RA NAD+ depletion is necessary and sufficient for poly(ADP‐ribose) polymerase‐1‐mediated neuronal death. J Neurosci. 2010; 30:2967-2978 20181594 10.1523/JNEUROSCI.5552-09.2010 PMC2864043

[48] Lyon AR Joudrey PJ Jin D Nass RD Aon MA O'Rourke B Akar FG Optical imaging of mitochondrial function uncovers actively propagating waves of mitochondrial membrane potential collapse across intact heart. J Mol Cell Cardiol. 2010; 49:565-575 20624394 10.1016/j.yjmcc.2010.07.002 PMC3081287

[49] Davidson SM Yellon D Duchen MR Assessing mitochondrial potential, calcium, and redox state in isolated mammalian cells using confocal microscopy. Methods Mol Biol. 2007; 372:421-430 18314743 10.1007/978-1-59745-365-3_30

[50] Grieshaber P Lagreze WA Noack C Boehringer D Biermann J Staining of fluorogold‐prelabeled retinal ganglion cells with calcein‐AM: a new method for assessing cell vitality. J Neurosci Methods. 2010; 192:233-239 20691729 10.1016/j.jneumeth.2010.07.037

[51] Song ZF Ji XP Li XX Wang SJ Wang SH Zhang Y Inhibition of the activity of poly (ADP‐ribose) polymerase reduces heart ischaemia/reperfusion injury via suppressing JNK‐mediated AIF translocation. J Cell Mol Med. 2008; 12:1220-1228 18782186 10.1111/j.1582-4934.2008.00183.x PMC3865666

[52] Braunwald E Kloner RA Myocardial reperfusion: a double‐edged sword? J Clin Invest. 1985; 76:1713-1719 4056048 10.1172/JCI112160 PMC424191

[53] Brustovetsky N Brustovetsky T Jemmerson R Dubinsky JM Calcium‐induced cytochrome c release from CNS mitochondria is associated with the permeability transition and rupture of the outer membrane. J Neurochem. 2002; 80:207-218 11902111 10.1046/j.0022-3042.2001.00671.x

[54] Liu RR Murphy TH Reversible cyclosporin A‐sensitive mitochondrial depolarization occurs within minutes of stroke onset in mouse somatosensory cortex in vivo: a two‐photon imaging study. J Biol Chem. 2009; 284:36109-36117 19892710 10.1074/jbc.M109.055301 PMC2794726

[55] Kerr PM Suleiman MS Halestrap AP Reversal of permeability transition during recovery of hearts from ischemia and its enhancement by pyruvate. Am J Physiol. 1999; 276:H496-H502 9950850 10.1152/ajpheart.1999.276.2.H496

[56] Zhao ZQ Velez DA Wang NP Hewan‐Lowe KO Nakamura M Guyton RA Vinten‐Johansen J Progressively developed myocardial apoptotic cell death during late phase of reperfusion. Apoptosis. 2001; 6:279-290 11445670 10.1023/a:1011335525219

[57] Choudhury S Bae S Ke Q Lee JY Kim J Kang PM Mitochondria to nucleus translocation of AIF in mice lacking Hsp70 during ischemia/reperfusion. Basic Res Cardiol. 2011; 106:397-407 21387140 10.1007/s00395-011-0164-1 PMC3205442

[58] Zischka H Larochette N Hoffmann F Hamoller D Jagemann N Lichtmannegger J Jennen L Muller‐Hocker J Roggel F Gottlicher M Vollmar AM Kroemer G Electrophoretic analysis of the mitochondrial outer membrane rupture induced by permeability transition. Anal Chem. 2008; 80:5051-5058 18510346 10.1021/ac800173r

[59] Formentini L Macchiarulo A Cipriani G Camaioni E Rapizzi E Pellicciari R Moroni F Chiarugi A Poly(ADP‐ribose) catabolism triggers AMP‐dependent mitochondrial energy failure. J Biol Chem. 2009; 284:17668-17676 19411252 10.1074/jbc.M109.002931 PMC2719406

[60] Zhang LQ Qi GX Jiang DM Tian W Zou JL Increased poly(ADP‐ribosyl)ation in peripheral leukocytes and the reperfused myocardium tissue of rats with ischemia/reperfusion injury: prevention by 3‐aminobenzamide treatment. Shock. 2012; 37:492-500 22266967 10.1097/SHK.0b013e31824989d7

[61] Nakajima H Kakui N Ohkuma K Ishikawa M Hasegawa T A newly synthesized poly(ADP‐ribose) polymerase inhibitor, DR2313 [2‐methyl‐3,5,7,8‐tetrahydrothiopyrano[4,3‐d]‐pyrimidine‐4‐one]: pharmacological profiles, neuroprotective effects, and therapeutic time window in cerebral ischemia in rats. J Pharmacol Exp Ther. 2005; 312:472-481 15466246 10.1124/jpet.104.075465

